# The equations of ametropia: Predicting myopia

**DOI:** 10.1016/j.optom.2021.08.001

**Published:** 2021-09-29

**Authors:** Francisco Gaya, Antonio Medina

**Affiliations:** a Massachusetts Institute of Technology, EE Research Laboratory, 77 Massachusetts Ave, Cambridge, Massachusetts 02139, United States; b Instituto de Investigación Hospital Universitario La Paz, Sección de Bioestadística, Paseo de la Castellana 261, Madrid 28046, Spain; c Multivision Research, 3106 N Commerce St., Stockton, California 95204, United States

**Keywords:** Myopia, Emmetropization, Cause of myopia, Myopia progression, Myopia theory

## Abstract

Why myopia develops, why it is reaching epidemic proportions and what is its cause are questions that puzzle many people. There is an answer to these questions and it is a simple one. This paper makes the connection between ametropic and in particular myopic development and theory to come with a summary of what we know about myopia and its governing equation.

Key experiments, involving myopia and the effect of lenses in humans and animals have been done with unmistakable results. The observed effect of lenses implies a feedback mechanism. Feedback theory explains those results with mathematical precision. Disruption of emmetropization, is the mechanism behind ametropia and particularly myopia.

Feedback theory for emmetropization was derived by observation of the input and output of the emmetropization feedback system in many patients. We show that it has the same equation as it is derived here independently from simple homeostasis principles.

Classical observations and recent clinical studies have shown the association of many variables with myopia. They include near work, atropine, lenses, blur and outdoors versus indoors activities. We propose that human refractive development is controlled by homeostasis and based on that alone we derive the equation for the calculation of refraction for any patient and the effect of lenses.

We provide software to calculate the refraction of any individual at any time.

The editor of this journal makes the following statement: “This manuscript is intended for scientific discussion rather than clinical application. The present work does not intend to promote clinical under correction or no correction of myopia. Instead, clinicians should follow current clinical myopia management guidelines."

## Introduction

### The connection between myopia and emmetropization

It is now universally accepted that there is a regulating mechanism controlling the refraction of the eye. The mechanism was called emmetropization and it is defined as the controlling process that regulates the refraction of the human eye to achieve optimal visual acuity over the years.^1^ Interference with emmetropization results in ametropia, most notably myopia.^2^^,^^3^

The mechanism that connects myopia with its cause has been elusive. Synthesizing the knowledge that we have about myopia to come with the prevailing myopia theory requires an enormous analysis of clinical and experimental data obtained in the latest decades. The processing of the data is not trivial as it requires advanced knowledge in several fields of science, including ophthalmology, physics, mathematics and engineering. To complicate things, many clinical and experimental studies were poorly designed and not surprisingly many published results are conflicting, contradictory or inconclusive, or contain unsupported conclusions.

Emmetropization is nothing more than refractive control by homeostasis. Its equation can be derived independently from homeostasis basic principles or by observation of refractive development in individuals corresponding to the output of a negative feedback system controlling the refraction of the eye. We show here that the results are the same using both independent methods.

### The basis of homeostasis

In natural sciences like physics and physiology, a variable not in equilibrium tends to reach a state of equilibrium. It is habitual to define the difference between the state of equilibrium and the current state as the stimulus or error.

A usual way or response to reach the equilibrium state is with a variation of the current state that is proportional to the error. The constant of proportionality k has the dimension of time. This physical observation, when applied to temperature differentials is known as Newton's law of cooling.

In all cases of basic control, the equation is the same: the variation of the current state is proportional to the error.

In physiology, a stimulus can be treated as an imbalance that is responded to. So, the metabolism of many administered substances, the size of an individual while in the same phase of growth and the muscle size adaptation to exercise also follow to some extent that model.

### Maintaining homeostasis

Biological variables in the human body are constantly being pushed away from their balance points or set points. For instance, during exercise, muscles increase heat production, pushing body temperature upward. Similarly, drinking sweet juice makes blood glucose go up. Homeostasis depends on the ability of the human body to detect and oppose these changes.

Maintenance of homeostasis usually involves negative feedback loops. These loops act to oppose the stimulus that triggers them. For example, if body temperature is too high, a negative feedback loop will act to bring it back down towards the set point, or target value, of 37.0 degrees C. The control center or feedback system will process the stimulus and respond with output activating effectors—such as the sweat glands—whose job is to oppose the stimulus by bringing body temperature down. The larger the error the greater the response.

**Fig. 1:**
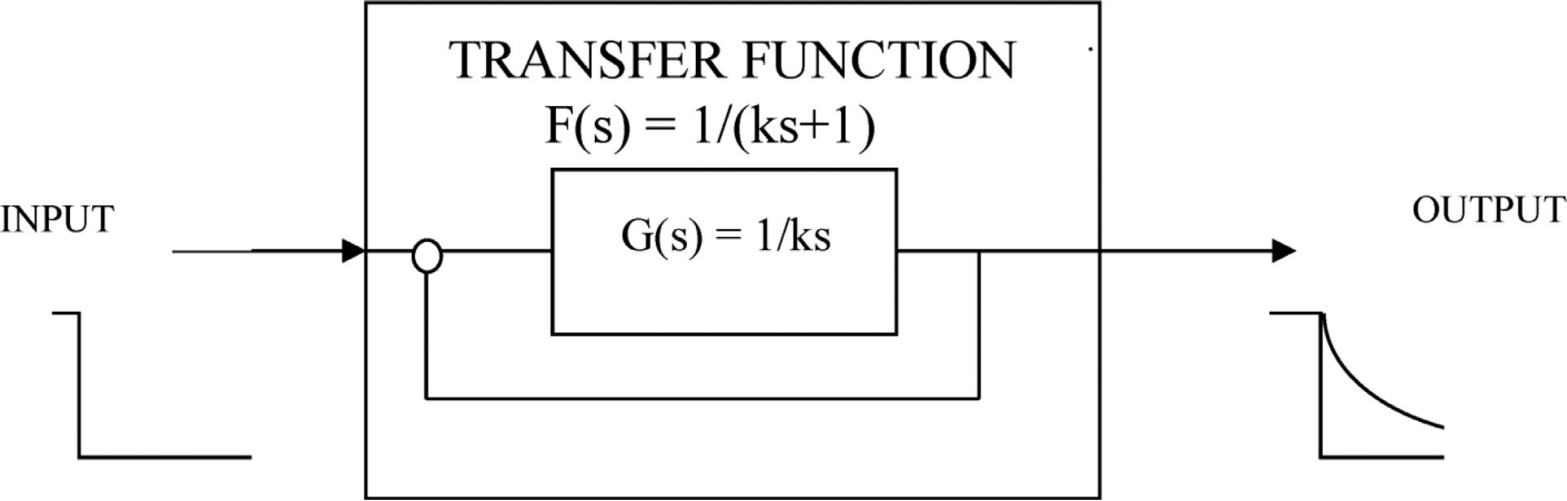
Feedback Theory transfer function F(s) = 1/(ks+1). This function can be derived by dividing the observed exponential refractive time course of individuals (1-e^−t/k^) by the step input in the complex domain. The exponential approach 1-e^−t/k^ transforms to 1/s(ks+1) and a step input to 1/s, so dividing them we obtain F(s) = 1/(ks+1). The exponential refractive output is observed in uncorrected individuals.

It was proposed that the shift towards and maintenance emmetropia is controlled by a second-order feedback system.^1^ The feedback system was simplified and a first-order was soon proposed when it was found from human data that an exponential function was a good fit.^4^ A first-order feedback system is a particular case of the second-order system, its transfer function is F(s)=1/(1+ks). See Fig. 1. This feedback function is termed here and in the literature as “Feedback Theory”, see eg.^5^,^6^ We also refer to it as “Proposed Theory“ or “Theory”. We derive here a mathematical equation based on refractive homeostasis and show that it is identical to the equation of Feedback Theory proposed in.^3^^,^^4^

## Methods

We use basic homeostasis law to model the progression with time of the refraction of the eye r(t), being the equilibrium state the set point (in diopters), and the stimulus is the error related to refractive error and includes the correction with lenses.

The natural refraction R for an individual is the set point  (R_0_) plus a near demand (d_n_) in diopters along intervals of time. The near demand is the average over that time of the equivalent lens that represents the use of near vision.^6^ The correction (g_n_) is the power (in diopters) of the lens worn by the patient over time and adds to the final equilibrium state that the eye is approaching.

(1) $$\text{dr}\left(t\right)/\text{dt}=\left[R+g_{n}-r\left(t\right)\right]/k$$

Solving this differential equation (see Appendix I.1), where the natural refraction R, and the correction g_n_ are constants, we have the equation of the progression of eye refraction:

$$r\left(t\right)-r\left(t_{n}\right)=\left[R+g_{n}-r\left(t_{n}\right)\right]\cdot \left(1-e^{-(t-t_{n})/k}\right)\text{with }t_{n}\leq t\leq t_{n+1}(2)$$

This equation corresponds exactly to the equation derived using Feedback Theory^4^:

$$r\left(t\right)=R+A\cdot e^{-t/k}+{\Sigma_{i=}}_{1}^{n}\left[\left(g_{i}-g_{i-1}\right)\cdot \left(1-e^{-\left(t-t_{i}\right)/k}\right)\right]\text{with }t_{n}\leq t\leq t_{n+1}(3)$$

if we work within time intervals between corrections, as demonstrated in Appendix I.2.

Feedback Theory explains and offers the equation to calculate the refraction of patients, including the changes caused by lenses. A vast number of observations support Feedback Theory.^6^ This report reviews the basic principles of homeostasis as applied to myopia and ametropia. Those principles show that Feedback Theory is not a complex theory without solid support, but quite the opposite as it can be derived from observation of refractive development and simple homeostasis principles. It explains with precision the effect of corrective lenses and the cause of myopia. Feedback Theory is the preeminent theory for emmetropization today because of its ability to explain a wide range of phenomena quantitatively. Two notable predictions of the Theory are described below.

## Results

**Fig. 2:**
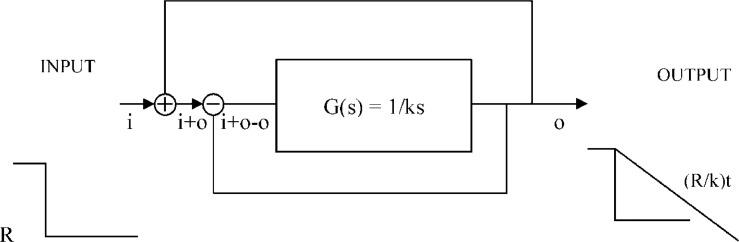
Negative lenses open the feedback loop. The feedback loop in Fig. 2 (lower loop) and the loop created by continuous correction (upper loop) cancel each other because i+o-o=i. The open-loop transfer function G(s) outputs a straight line in time and keeps myopia advancing at a rate of R/k.

Feedback Theory predicts, as it has been observed, that a myope who starts wearing minus lenses will fall in an uncontrolled myopia advance of no return.^3^ The same will happen to a non-myope that uses his eyes massively for near work as that is equivalent to wearing a minus lens. See Figs. 2 and 4.

**Fig. 3:**
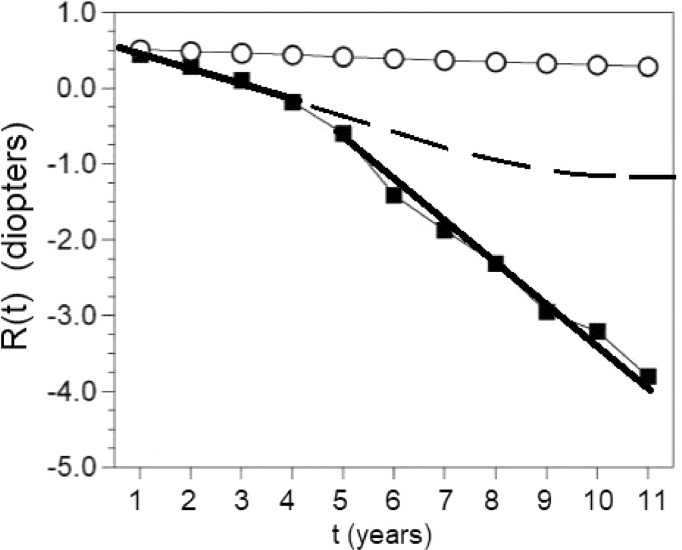
The fall into the myopia depression. The average refractions of children who became myopic are fitted with straight thick lines before (years 1 to 4) and after correction (years 5 to 11). Notice the change in the rate of myopia progression. The slope of the line triples from -0.18D/y to -0.54D/y after they are corrected. Uncorrected children (circles), subjected to the same environmental conditions do not fall into the myopia depression. If the children had not been corrected we calculated with the equation described here that their myopia would have stabilized at an estimated average of 1.12D (broken trace). Redrawn from data in^5^ with regression lines, time axis labels, and Feedback Theory prediction curve added.

**Fig. 4:**
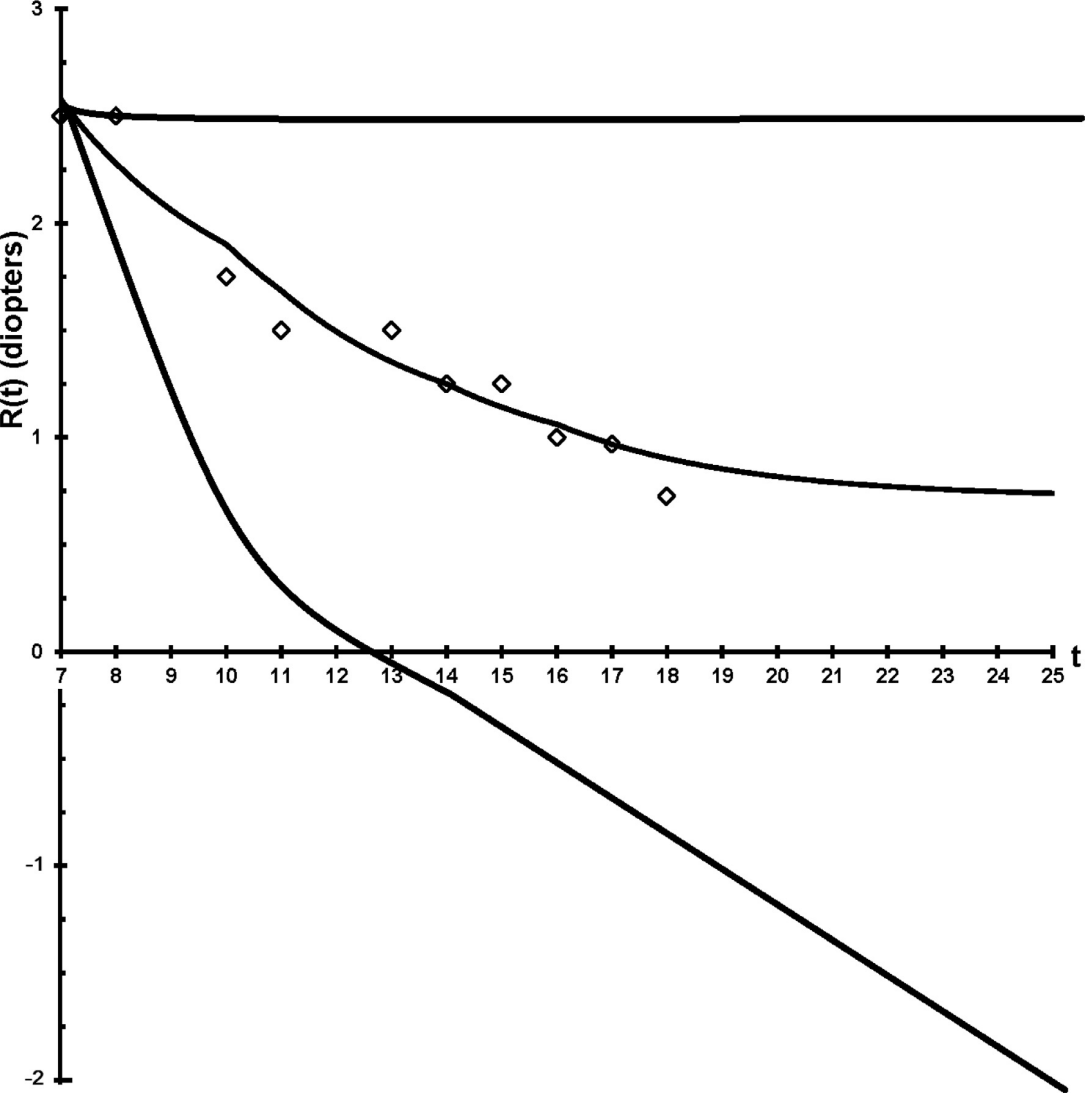
Depiction of how the choice of lenses used since age 7 can alter the refraction at age 25 within the range +2.5D to -2.5D. Actual refractive development (diamonds, spherical equivalent) of a child's eye whose hyperopia was corrected by 50% from age (t) 7 to 17 years and prediction of Feedback Theory if such correction were maintained indefinitely (R(t), middle trace). The designed lens treatment made this child emmetropic. Flat prediction if the same child's hyperopia had been fully corrected at age 7 (upper trace). Prediction for an alternative treatment: the same child's hyperopia had not been corrected, he had an increased near demand of 1D and his myopia had been fully corrected every time it increased by 0.25D. All predictions were made using equations in this report. Drawn from the author's data.

### The effect of correcting myopia

The feedback error may be zero for a refractive error near emmetropia. When there is a myopic refractive error, and we place a lens “correction” over the eye emmetropization will lengthen the eye to regain its target myopia, normally another more powerful lens is prescribed again and again. The emmetropization feedback loop is opened and the response is a steep linear progression of myopia as derived here in the time domain and elsewhere in the complex domain.^3^ The steep decline and eventual stabilization floor of this uncontrolled progression is termed the “myopic depression”.^3^, ^6^ See Figs. 2Fig. 3Fig. 4.

### Near work

Near work, meaning near vision of any kind including viewing electronic displays is associated with myopia.^8^, ^9^, ^10^, ^11^, ^12^, ^13^, ^14^, ^15^, ^16^, ^17^, ^18^, ^19^, ^20^, ^21^, ^22^, ^23^, ^24^ Near work is equivalent to negative lenses based on the principle of equivalence^2^, ^6^ and therefore causes myopia according to Feedback Theory.

The tremendous increase in the prevalence of myopia in recent years is probably due to the high myopigenic effect of small display screens used in modern telephones and other electronic devices. For example, common phone displays with close to 500 pixels per inch (ppi) require a viewing distance of about 33cm, and ultra-high density screens of 800ppi require a distance of about 20cm, calculated by dividing the 2-pixel distance by the eye angular resolution. These reduced viewing distances are equivalent to lenses of -3 or -5 diopters placed in front of the eye, which, as discussed here, cause myopia.

### Other results or corollaries from the equations in this report

The given equations are easier to analyze if we assume from the beginning that they can only supply information in a refractive period with a lens correction. The refractions are numbered by ordinal numbers. The periods are numbered as their starting refraction. To simplify:

$$\Delta t_{n}=t_{n}-t_{n-1}$$

$$\Delta r_{n}=r\left(t_{n}\right)-r\left(t_{n-1}\right)$$

$$u_{n-1}=r_{n-1}-g_{n-1}\left(\text{if full correction, }r_{n}=g_{n},\text{undercorrection }u_{n}=0\right)$$

The equation then, applied to the i-th refraction, is denoted by:

$$\Delta r_{n}=\left(R-u_{n-1}\right)\cdot \left(1-e^{-{\mathrm{\Delta t}}_{n}/k}\right)$$

If the near demand and the correction are constant in the periods between reliable and accurate refractions:

1. The change in refractive error after a clinical refraction is performed is a function only of the time and the correction.
2. If in two consecutive refractions with Δt_n_ ≠ 0, refraction remains constant (Δr_n_=0), then the system has reached an equilibrium state, and R = u_n-1_.
3. The equation can be transformed to:
  $$-\Delta t_{n}/k=\text{ln}\left[1-∆r_{n}/\left(R-u_{n-1}\right)\right]$$
  As both k and Δt_i_ have limits: 0 < k <∞, 0 < Δt_i_ <∞,
  $$-\infty <\text{ln}[1-\Delta r_{n}/\left(R-u_{n-1}\right)]<0$$
  and then
  $$1>\Delta r_{n}/\left(R-u_{n-1}\right)>0$$
  so, the relation between Δr_n_ and (R–u_n-1_) is quite precise:
  $$\text{sign}\left(\Delta r_{n}\right)=\text{sign}\left(R-u_{n-1}\right)$$
  $$\left|{\mathrm{\Delta r}}_{n}\right|<\left|R-u_{n-1}\right|$$
4. If the duration of every time period between refractions is equal, and has constant correction (Δt_n_ =T, u_n-1_ = u ⇒ Δr_n_=C), the slope m, or rate of myopia progression, is constant and
  (A) $$m=C/T=\left(R-u\right)\cdot \left(1-e^{-T/k}\right)/T$$
  Approximating with McLaurin's series:
  (B) $$m=\left(R-u\right)\cdot \left(1/k-T/2k^{2}+T^{2}/6k^{3}-\ldots \right)$$
  So, if T/k < 3 (and better approximation as T decreases)
  m≥(R−u)/k
5. The slope is less as the time between corrections T increases. That is, myopia progresses more slowly when corrections are less frequent. In the case of continuous (or very frequent) correction, T tends to 0

Taking the limit of eq (A) using l'Hopital's rule, or simply substituting T = 0 in equation (B):
  m=(R−u)/k
If full correction (u = 0) then:
  m=R/k
As derived by Medina analytically from Feedback Theory in the complex domain.^3^

## Discussion

It is generally accepted that the visual environment is a major contributor to school-aged myopia. Additionally, the risk of myopia development and progression is significantly associated with reading at very close distances.^24^ Several factors are associated with myopia such as near work, indoor activities, power of lenses used, and others discussed here. They have one thing in common, negative lenses. The association not only supports this Theory but is independent evidence of the cause of myopia. That negative lenses affect myopia is not a surprising finding, the belief that they lead to accelerated progression of myopia has been reported frequently.^1^, ^2^, ^3^, ^4^^,^^6^^,^^25^, ^26^, ^27^, ^28^

It can be inferred from a combination of studies that correction of myopia increases it, as Feedback Theory predicts, even though that was not the tested hypothesis. See e.g.^29^^,^^30^. Feedback Theory predicts that the frequency distribution for a group of uncorrected adults would be leptokurtic with no myopia skew and that myopia and its prevalence should be low. A study of over 10000 adults (>40y) Nigerian subjects with myopia (< -0.5D and no cataracts) prevalence of 10.1% showed that these myopes were mostly uncorrected (98.8% wore no distance spectacles) and about 75% had a refractive error between 0 and -1D.^29^ A comparable group of about 6000 Californian urban blacks of the same age had myopia (< -1D) prevalence of 29%,^30^ the prevalence of high myopia (-5.0D) was 5.1%, vs. 0.7% in the Nigerian group. All these data confirm the prediction. Since the American group was surely corrected, those studies provide compelling evidence that correction is causative of myopia. Analysis of data from other studies shows that correction of myopia, including near work, results in increased myopia.^31^, ^32^, ^33^, ^34^

A test of Feedback Theory designed to show the effect of corrective lenses would lead to changes in the management of myopia and mass prevention. Medina made some suggestions for the design of such a study, using the same subjects to evaluate the myopia progression before and after correction.^35^ Similar proposals can be found in the literature.^27^

### General limitations

Although feedback control is the preeminent model for myopia today because of its ability to explain a wide range of phenomena, such as school myopia or the effect of atropine,^36^, ^6^ Feedback Theory does not account for axial elongation and considers only refraction. This model considers only the refractive history and does not takes into account the age or other relevant factors that might be relevant to explain the onset, development, and stabilization of myopia. Although the feedback system can reverse myopia^37^, ^38^, ^39^, ^40^, ^41^, ^42^ it is unknown whether feedback is operational after a certain age.

### Limitations of the equations and particular cases without a solution

The simplified equation, applied to the i-th period, is:

$$\Delta r_{n}=\left(R-u_{n-1}\right)\cdot \left(1-e^{-{\mathrm{\Delta t}}_{n}/k}\right)$$

The near demand and the correction must remain constant for each interval between reliable and accurate refractions. If the near demand changes, it must be known and taken into account, using the equation:

$$\Delta r_{n}=\left(R_{0}+d_{n-1}-u_{n-1}\right)\cdot \left(1-{e^{-}}^{\mathrm{\Delta t}n/k}\right)$$

To solve the unknown parameters (R and k, or R_0_ and k, if we know d_i_ and changes between intervals), we need at least 2 equations (*i* and *j*). As each equation refers to a period between refractions, we need at least 3 refractions. As expected, with more refractions, the calculation error of the parameters diminishes.

Every time interval between periodic refractions, that is equally timed and with constant correction (Δt_n_ = T, u_n-1_ = U), must produce exactly the same values for the equation, and cannot be used to find the parameters R and k.

The given equation is transcendental, so solving it analytically is not feasible except if:

1. Periodic refractions are equally timed (Δt_n_ = T) with different under correction (as this is necessary to obtain different values for Δr_i_ and Δr_j_):
  $$R=\left(u_{i-1}\cdot \Delta r_{j}-u_{j-1}\cdot \Delta r_{i}\right)/\left(\Delta r_{j}-\Delta r_{i}\right)$$
  $$k=-T/\text{Ln}[1-\left(\Delta r_{i}-\Delta r_{j}\right)/\left(u_{j-1}-u_{i-1}\right)]$$
2. The time of one interval is exactly the double of the other interval (Δt_i_ = 2Δt_j_), it is possible to simplify to a second-degree equation of the exponential, but results in a convoluted expression except if under correction is in both cases 0:
  $$R=\Delta {r_{j}}^{2}/\left(2\cdot \Delta r_{j}\Delta r_{i}\right)$$
  $$k=-\Delta t_{j}/\text{Ln}\left(\Delta r_{i}/\Delta r_{j}-1\right)$$

In general, as two intervals are too few to get good accuracy of prediction, using numerical methods (ie. Newton-Raphson) is the only practical way to find the value of the parameters R and k.

## Conclusions

Many conclusions can be reached using Feedback Theory. It predicts that a myope who starts wearing minus lenses full time with the full prescription will fall into a myopic depression of no return.^3^ Myopia is also the result of near work. As all that happens, nothing will stop myopia.

**Fig. 5:**
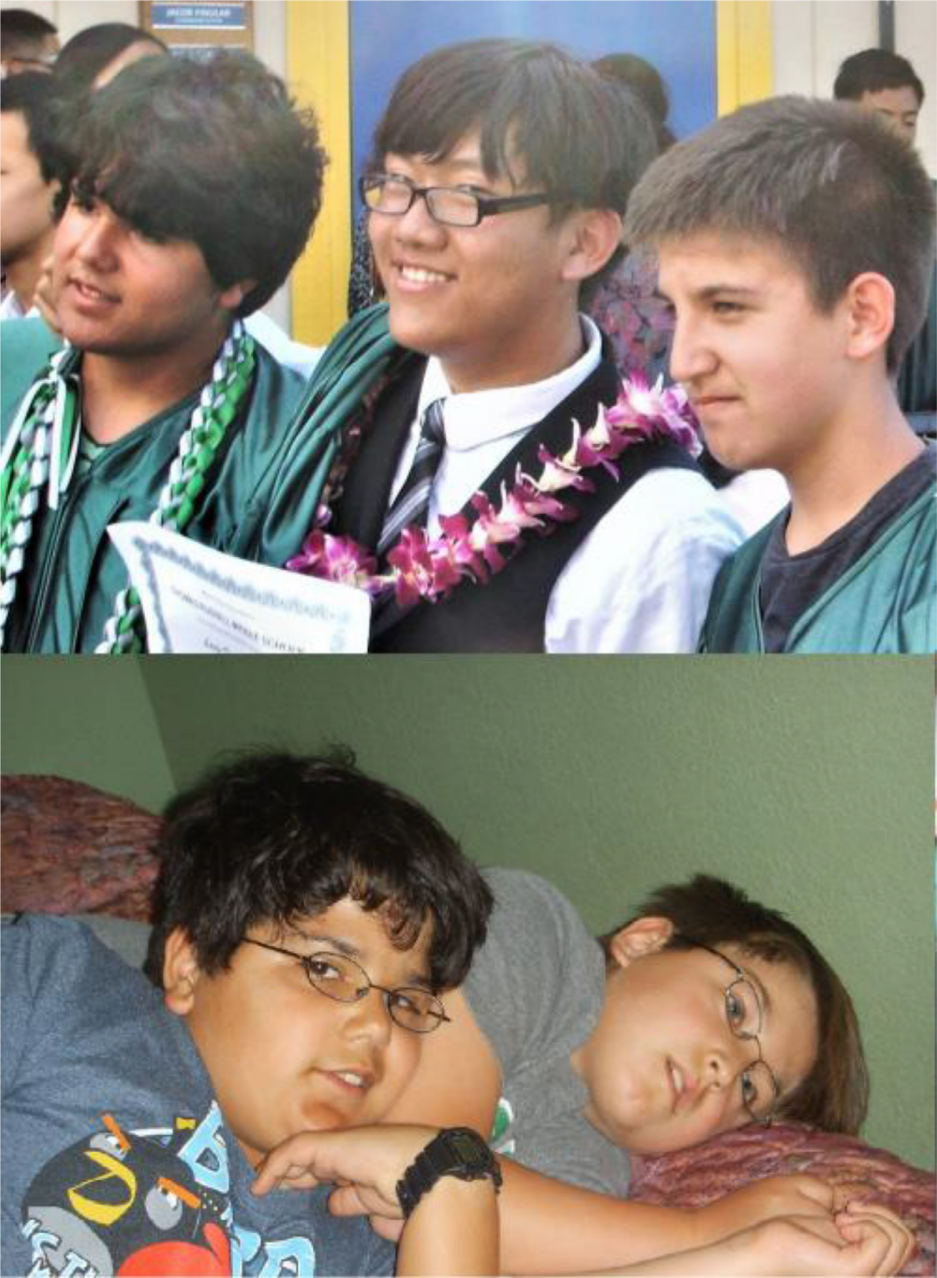
The Theory was put to the test to prevent myopia in an 11-year trial that culminated in 2020. These two boys in green gowns were at risk of developing myopia, but are free of myopia and glasses at graduation (upper photo) after 10 years of preventive positive lens wear (lower photos). Just as Feedback Theory predicted. See^44^ and Fig. 4 for details of the trial.

The current prescription and use of lenses simply to achieve the best visual acuity can be changed to smart prescriptions designed with the equations here to lessen refractive error or prevent myopia.^43^^,^^44^ See Figs. 4 and 5. Less frequent correction of myopia will result in a slower progression rate as derived here.

Those treatments for myopia and others are not only suggested here but some have been successful already.^44^ Feedback Theory predicts that if myopia is not corrected, or under corrected at least R diopters, it would stabilize, while it will progress linearly when corrected. Myopia is not a disease that can be cured because it is not a disease. This is what we can do: arrest and slow its progression and prevent it in the first place.

## Conflict of Interest

The authors declare that they have no conflict of interest.

## Figures and Tables

**Table:** 

○ h_VNn_	(Very Near focus)	∼	–4 diopters = ¼m:	tablets, mobilephones or books
○ h_Nn_	(Near focus)	∼	–2 diopters = ½m:	using PC
○ h_Mn_	(Medium focus)	∼	–½ diopters = 2m:	indoors, conversation with people, television at normal distance…
○ h_Fn_	(Far focus)	∼	–0 diopters > 2m:	outdoors

## Appendix I

### I.1 Solution of the differential equation representing refraction with time, by integration

The differential equation of refraction r(t), as justified in the text, is:

(1) $$\text{dr}\left(t\right)/\text{dt}=\left[R+g_{n}-r\left(t\right)\right]/k$$

R and g_n_ are constant over the period n, so dR/dt = 0 and dg_n_/dt = 0. Subtracting both (they are 0) from the left side, and changing sign in the right:

$$d\left[r\left(t\right)-R-g_{n}\right]/\text{dt}=-\left[r\left(t\right)-R-g_{n}\right]/k$$

$$d\left[r\left(t\right)-R-g_{n}\right]/\left[r\left(t\right)-R-g_{n}\right]=-\text{dt}/k$$

Integrating for any t inside the period n so that t = [t_n,_ t]:

$$r\left(t\right)-R-g_{n}=\left[r\left(t_{n}\right)-R-g_{n}\right]\cdot e^{{}^{-(t-t_{n})/k}}$$

(2) $$r\left(t\right)-r\left(t_{n}\right)=\left[R+g_{n}-r\left(t_{n}\right)\right]\cdot \left(1-e^{-(t-t_{n})/k}\right)$$

If no further correction is done, the refraction asymptotically approaches

$$r\left(\infty \right)=R+g_{n}\text{if }t=\infty$$

### I.2 Proof of the equality of Feedback Theory and homeostatic adaptive response over time

Feedback Theory is defined by its transfer function F(s) = 1/(ks+1). The transfer function is defined in the complex domain, where s is the complex variable and k is the time constant. Input and output can be represented in the time domain or complex domain. This function was derived by dividing the observed output exponential refractive time course (1-e^−t/k^) by the step input, both in the complex domain. The exponential approach 1-e^−t/k^ transforms to 1/s(ks+1) and a step input to 1/s in the complex domain, so dividing them we obtain F(s) = 1/(ks+1). A step, such as a lens of power R, is the input that causes the corresponding exponential refractive output.

Since different corrective lenses are applied many times during different periods, the overall refraction of a patient is the summation of many exponential functions^4^. In sigma notation equation 4 in^4^ is:

$$r\left(t\right)=R+A\cdot e^{-t/k}+\sum_{i=1}^{n}\left[\left(g_{i}-g_{i-1}\right)\cdot \left(1-e^{-(t-t_{i})/k}\right)\right]\text{with }t_{n}\leq t\leq t_{n+1}(3)$$

Applying Eq. (3) to the moment the period begins, t_n_, and multiplying both sides by e^−(t^^−^^tn)/k^:

$$r\left(t_{n}\right)\cdot e^{-(t-t_{n})/k}=R\cdot e^{-(t-t_{n})/k}+A\cdot e^{-t/k}+\sum_{i=0}^{n}\left[\left(g_{i}-g_{i-1}\right)\cdot \left(e^{-(t-t_{n})/k}-e^{-(t-t_{i})/k}\right)\right]$$

Subtracting the previous equation from the Eq. (3), and as g_0_=0:

$$r\left(t\right)-r\left(t_{n}\right)\cdot e^{-(t-t_{n})/k}=\left(R+g_{n}\right)\cdot \left(1-e^{-(t-t_{n})/k}\right)$$

Operating:  

(4) $$r\left(t\right)-r\left(t_{n}\right)=\left(R+g_{n}-r\left(t_{n}\right)\right)\cdot \left(1-e^{-(t-t_{n})/k}\right)$$

Eq. (4) is identical to Eq. (2) showing that both approaches are equivalent.

## Appendix II The near demand and correction

To calculate the set point R_0_ we propose using the near demand (d_i_) and correction (g_i_) for simplicity. We need g_i_ because different correction may be used for diferent distances.

Since there is evidence that emmetropization works only with active vision, both near demand and correction average must be calculated over waketime (averaged daily) h_Ai_. It consists in both cases of a vector product of the percentage of time awake focusing a certain distance for near demand or diopters of the lens for correction.

The differences in distance could be grouped with no great loss of precision in the average daily time by focus distance by groups, for the n-th period:

So, using this groups, the average accommodation would be, for the n-period:

$$d_{n}=\left(-4\cdot h_{\text{VNn}}-2\cdot h_{\text{Nn}}-1/2\cdot h_{\text{Mn}}\right)/h_{\text{An}}$$

We can then calculate R_0_ from the equation:

$$R=R_{0}+d_{n}$$

We could proceed in the same way if we know, for period n, the diopters of each i-th lens used (c_ni_) and its percentage of use over waketime for the nth-period (p_ni_):

$$c_{n}=\Sigma_{i}\left(c_{\text{ni}}\cdot p_{\text{ni}}\right)$$

## Appendix C Supplementary materials

Image, application 1
