# Supplementary material for: The equations of ametropia: Predicting myopia
Source: J Optom. 2021 Sep 29;15(3):238–46. doi: 10.1016/j.optom.2021.08.001 (PMC9237633; doi:10.1016/j.optom.2021.08.001)
Supplement: Supplementary file 1 [file mmc1.pdf]

SUPPLEMENTAL MATERIAL

A numerical calculator is provided that can estimate and graph the refraction of any eye given 5 refractions and corresponding corrections.

INSTRUCTIONS TO DOWNLOAD AND RUN THE EYE REFRACTION PROGRESS CALCULATOR

Get the calculator:

1. Ctrl-Click on the link below or copy the direction in your web browser.
   https://drive.google.com/drive/folders/1vYeRBWS3qsBLIewUDJKAuXOxY5S-jm5f?usp=sharing
2. Download the file "Eye Refraction Progress Calculator.zip" Right-click on it and choose the Download option from the drop-down menu.
3. Open in your equipment the zip file and the .xls file it contains.

The program requires that a version of Microsoft Excel is installed in the computer as it uses it for graphing. Click "Enable Editing" if you are executing in protected view.

How to use the calculator:

The calculator accepts 5 refractions at 5 dates plus the corrections prescribed and worn. These data are input in the indicated boxes, the earliest refraction goes on top and successive ones below in chronological order. It will take any refraction spherical equivalent, myopic, emmetropic or hyperopic and any correction. For full correction the fields for refraction and correction must be the same. The "Process Data" button must be clicked to update the graph. The graph will display the data, the refraction prediction and the prediction without any correction. The time span graphed is about 16 years. The calculator can provide "what if'' predictions for any under correction or no correction as well as any near demand equivalent power or plus lens used.

Near demand, if known or estimated, must be added to the correction; for example, a hyperope of 2D fully corrected and accommodating an average of 1.5D (near demand) has a correction of 2-1.5=0.5D, an emmetrope with the same near demand has a correction of -1.5D, a myope of -1D with the same near demand has a correction of -2.5. The last correction input can be anything to make "what if'' predictions.

This is a beta version, intended for demonstration. It can also be used with data in this paper and similar data for calculations and predictions. It must be used with actual refraction data. Using this program with assumed data will not necessarily result in assumed results. Do not use this program to validate your assumptions. Errors in the data will result in substantial errors in the prediction. Do not use this program with refractions of dubious accuracy or non-cycloplegic refractions in the case of hyperopes. Use this program only if the correction worn by the patient is known as well as the date. Do not assume that the correction was worn at all times. This program assumes, for simplicity, that the date of refraction and date of correction are the same. If the date of refraction and date of correction are not substantially the same use the date of correction for both. Do not use this program if the refractions are equally spaced in time unless the under corrections are different.

The calculator software is based on the equations for the first order feedback system as defined in this paper and the references below after mathematical treatment, simplification and computer optimization by F. Gayá. A. Medina designed the numerical method used to calculate the solution.

REFERENCES

Medina A. The progression of corrected myopia. Graefes Arch Clin Exp Ophthalmol. 2015;253(8):1273-1277.
Medina A. The cause of myopia development and progression: Theory, evidence, and treatment. Survey of Ophthalmology (2021), 10.1016/j.survophthal.2021.06.005
Medina A and Fariza E. Emmetropization as a first-order feedback system. Vision Res. 1993;33(1):21-26.

USE OF THE SOFTWARE

1) The software “Eye Refraction Progress Calculator” or (“Software”) is a program developed for the readers of the Journal of Optometry. This Software is part of the publication titled: “THE EQUATIONS OF AMETROPIA: PREDICTING MYOPIA” in the Journal of Optometry. You can freely install and use this Software for scientific and educational purposes. However, to use this Software at business companies, clinics, any public offices, or other locations serving patients or the public at large, you must obtain permission from the authors of the paper. You may not use this Software for profit-seeking commercial services whether directly or indirectly, or as any part thereof.

2) You may use this Software and its related graphs and documents strictly in accordance with the statutes concerning intellectual properties and all of the other relevant laws of the region where you use this Software.

3) You may not use this Software in connection with any device or software that is designed for the purpose of avoiding technical actions taken for protecting the content or other works (or rights to such use thereof) under the copyright law of the region where you use this Software.

4) Because this Software is protected under the Copyright Act and the Computer Software Protection Act, you may not modify, reverse-engineer, decompile or disassemble it beyond the limited scope explicitly allowed by such relevant laws.

5) Beta version releases of this Software are subject to changes in its functions, specifications, performance, characteristics, and other details, without any prior notice. In addition, beta versions of this Software may have errors that might affect its normal use. Installing any beta version of this Software shall mean that you accept the possibility of such occurrences.

6) Installing the Software shall mean that you have read, understood, and consented to these terms.
